# Gender differences in awareness and practices of cancer prevention recommendations in Korea: a cross-sectional survey

**DOI:** 10.4178/epih.e2025003

**Published:** 2025-01-15

**Authors:** Yoonjoo Choi, Naeun Kim, Jin-Kyoung Oh, Yoon-Jung Choi, Bohyun Park, Byungmi Kim

**Affiliations:** 1 Division of Cancer Prevention, National Cancer Control Institute, National Cancer Center, Goyang, Korea; 2 Department of Cancer Control and Population Health, Graduate School of Cancer Science and Policy, National Cancer Center, Goyang, Korea; 3 Division of Cancer Control and Policy, National Cancer Control Institute, National Cancer Center, Goyang, Korea

**Keywords:** Cancer, Primary prevention, Awareness, Practice, Sex factors, Korea

## Abstract

**OBJECTIVES:**

Gender is a major determinant of health behaviors that influences cancer prevention awareness and practices. This study investigated the relationship of the awareness and practice rates of cancer prevention recommendations with gender and socioeconomic status.

**METHODS:**

We used data from the Korean National Cancer Prevention Awareness and Practice Survey (2023). The sample included 4,000 men and women aged 20-74 years. We conducted multiple logistic regression analyses to evaluate associations with the awareness and practices of cancer prevention, and a joinpoint regression analysis using age-standardized rates to analyze trends in awareness and practice rates from 2007 to 2023.

**RESULTS:**

The awareness rates were 79.4% and 81.2% for men and women, respectively. The overall practice rates were substantially lower (43.1% for men and 48.9% for women). For men, awareness rates did not differ significantly by socio-demographic characteristics, but practice rates increased with age (20-29: 15.9%; 60-74: 53.8%). For women, both awareness (20-29: 73.0%; 60-74: 85.7%) and practice (20-29: 16.8%; 60-74: 67.5%) rates increased with age. The easiest recommendations to follow were “reducing salt intake and avoiding burnt or charred foods” (men: 29.9%; women: 28.4%), whereas the most difficult recommendation was “engaging in regular physical activity” (men: 32.5%; women: 34.4%).

**CONCLUSIONS:**

While awareness of cancer prevention recommendations was high, the practice of these recommendations was low. Gender influenced changes in awareness and practice rates over time, reflecting a large gap in practice. Future research should explore appropriate intervention points for cancer prevention practices and the development of more effective cancer prevention policies.

## GRAPHICAL ABSTRACT

[Fig f4-epih-47-e2025003]

## Key Message

Many factors influence cancer prevention awareness and practice, and gender is a crucial determinant of health behaviour in general. This study found that while awareness was reasonably high among both men and women, there was a significant gender gap in practice. Given the variation in health behaviours by gender, further evaluation of gender effects is necessary to inform future policy interventions aimed at improving cancer prevention practices.

## INTRODUCTION

Cancer is the second leading cause of death worldwide, killing 10 million people each year and resulting in 19.3 million new cases annually [1].

An unhealthy lifestyle is the most significant risk factor for cancer and is directly linked to global health issues; therefore, primary prevention is crucial not only for preventing cancer but also for addressing these health risks [2-5]. Various countries have developed and disseminated cancer prevention guidelines that focus on primary prevention, aiming to closely identify factors influencing cancer development and provide recommendations to mitigate these risks [6-8]. Similarly, the National Cancer Center Korea established and distributed its cancer prevention guidelines in 2006, which were revised in 2016. It currently maintains the Korean national recommendations against cancer [8], which are as follows:

(1) no smoking and avoiding secondhand smoke, (2) consuming a sufficient amount of fruits and vegetables and a balanced diet, (3) reducing salt intake and avoiding burnt or charred foods, (4) limiting alcohol consumption, (5) engaging in regular physical activity (i.e., at least 30 minutes, 5 days a week), (6) maintaining a healthy body weight, (7) immunization against hepatitis B virus (HBV) and human papillomavirus (HPV), (8) engaging in safe sex (i.e., having a single sexual partner and using condoms), (9) following all health and safety instructions aimed at preventing exposures to cancer-causing agents in the workplace, and (10) undergoing regular cancer screening.

For these recommendations to be effective, they must be fully understood and embraced by the public, translating into actual behaviors [9]. However, adopting cancer-prevention behaviors can be challenging due to numerous influencing factors. Among these, gender stands out as a significant determinant of health behavior [10-13]. The World Health Organization (WHO) has highlighted the importance of empirical data on gender differences and the necessity for gender mainstreaming efforts to enhance overall health [14]. Therefore, it is to acknowledge potential differences in cancer prevention awareness and practices based on gender. In this study, we examined the rates of awareness and practice of cancer prevention recommendations across genders and investigated how these rates vary with socioeconomic status.

## MATERIALS AND METHODS

To provide a basis for effective cancer prevention policies, this study analyzed data from the 2023 National Cancer Prevention Awareness and Practice Survey, conducted by the National Cancer Center, Korea. The sample was randomly selected and proportionally allocated according to the number of individuals by gender, age, and residential area, as per the resident registration demographics provided by the Ministry of the Interior and Safety of Korea. The survey was conducted face-to-face using a structured questionnaire. Participants voluntarily joined the survey after reviewing a recruitment guide that outlined the study’s purpose and content. The survey continued until the sample size reached 4,000 participants. Over a 49-day period from September 7, 2023 to October 25, 2023, a total of 22,074 respondents were approached. Of these, 9,102 (41.2%) declined to participate, 8,159 (37.0%) were not present during the survey visit, 599 (2.7%) did not meet the age requirements, and 214 (1.0%) also declined to participate. The final sample consisted of 4,000 participants, including 2,027 men and 1,973 women, aged 20-74 years, from various regions across the country. Additionally, data from previous surveys were used to analyze trends over time. The initial survey was conducted in 2007 and has been repeated biennially. Each survey was a cross-sectional study, with sample sizes determined based on the population proportions of region, gender, and age groups, followed by random sampling. The data collected from the surveys conducted between 2007 and 2023 were utilized for analysis in this study.

In this study, we utilized a structured questionnaire to assess public awareness and adherence to the Korean national recommendations against cancer, as established by the National Cancer Center. The survey was an updated version of the original created in 2007. The initial questionnaire was grounded in the precaution adoption process model (PAPM), a theoretical framework designed to elucidate how individuals adopt behaviors to prevent diseases, injuries, or other harm. This model is predicated on the idea that individuals progress through 6 distinct stages from unawareness to action. According to PAPM, these stages of preventive health behavior include: (1) being unaware of the health behavior, (2) not engaging in the decision-making process, (3) being undecided, (4) deciding against action, (5) deciding to take action, and (6) implementing the action. For the purposes of this study, the PAPM stages were grouped into 3 categories: (1) pre-adoption (stages 1-3), (2) rejection (stage 4), and (3) adoption (stages 5-6). To evaluate general awareness about cancer prevention, participants were asked, “Do you think cancer is preventable?” Subsequently, to assess specific practices related to cancer prevention, we inquired, “Have you made any specific efforts to prevent cancer?”

We conducted the chi-square test and cross-tabulation analysis based on gender to establish the general characteristics of the survey participants. Additionally, we performed multiple logistic regression analysis to determine the association between awareness and cancer-prevention practices. Odds ratios (ORs) and 95% confidence intervals (CIs) were calculated, taking into account correction variables such as gender, age, education level, and income quintile. Age was divided into 10-year increments, with the exception of those over 60 years old. Residential areas were classified as urban, mid-sized, or rural, while marital status was categorized as single, married, or widowed/separated/divorced. Education levels were grouped into 3 categories: middle school or less, high school graduate, and college graduate or higher. Monthly income was divided into 3 ranges: less than 3 million Korean won (KRW; less than US dollar [USD] 3,900), 3-6 million KRW (USD 3,900-7,800), and more than 6 million KRW (USD 7,800 or more). The age-standardized rates for cancer prevention awareness and practice for each gender were calculated based on the total population figures published by Statistics Korea in 2023, the same year the survey was conducted. To examine the trends in awareness and practice rates from 2007, when the survey was first conducted, to the present, we utilized joinpoint regression analysis with age-standardized rates. All statistical analyses were conducted using R version 4.1.3 (R Foundation for Statistical Computing, Vienna, Austria) and Joinpoint version 5.0.2 (National Cancer Institute, Bethesda, MD, USA) software. Statistical significance was established at p-value< 0.05.

### Ethics statement

The survey protocol and secondary use of the data were approved by the Institutional Review Board of the National Cancer Center, Korea (NCCNCS-07-102, NCC2016-0153, NCC2022-0012). All participants provided informed written consent before participating in the study.

## RESULTS

This study included 4,000 adult men and women aged 20-74, with 2,027 men (50.7%) and 1,973 women (49.3%) (Table 1). The most represented categories for both genders included individuals aged 60-74, residing in mid-sized cities, married, holding at least a college degree, and earning a monthly income of 3-6 million KRW.

Both genders demonstrated high overall awareness levels, with 79.4% for men and 81.2% for women. However, the rates of actual cancer prevention practices were less than 50% for both men and women (43.1% for men and 48.9% for women). The likelihood of engaging in these practices increased with age; the youngest group, those in their 20s, had the lowest rates (15.9% for men and 16.8% for women). This trend aligns with previous studies indicating that younger individuals often take greater health risks and tend to adopt healthier behaviors as they grow older [5,10,15]. Rural residents, typically living in areas with low population density and a focus on primary industries, showed the highest engagement in cancer prevention practices (46.8% for men and 57.2% for women). In contrast, residents of medium-sized cities had the lowest practice rates (40.2% for men and 45.8% for women), despite having the highest awareness levels. This discrepancy suggests that the physical and social environments significantly influence health behaviors, as noted in the literature [16,17]. Understanding why residents of medium-sized cities do not engage in cancer prevention practices, despite high awareness, is crucial. Married individuals were more likely to engage in prevention practices (50.5% for men and 57.1% for women), compared to their single counterparts (27.9% for men and 27.4% for women). Notably, single women practiced prevention at less than half the rate of married women (57.1%), and the rates for widowed, separated, or divorced individuals were slightly lower (56.5%).

**Figure 1.**
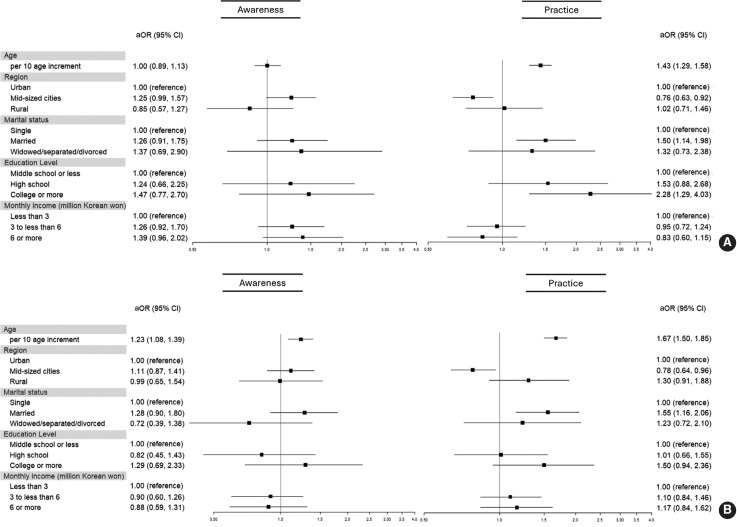
Adjusted odds ratios (aORs) and 95% confidence intervals (CIs) of related factors of awareness and practice of cancer prevention (A) men, (B) women. aORs were adjusted for age, residential area, marital status, education level, and monthly income. The x-axis represents the odds ratio on a logarithmic scale.

The ORs associated with the awareness and practice of cancer prevention are depicted in Figure 1. For men (Figure 1A), awareness rates were consistent across socio-demographic characteristics, whereas practice rates increased with age (adjusted OR [aOR], 1.43). Men residing in medium-sized cities demonstrated lower practice rates compared to those in urban areas (aOR, 0.76). Married men were more likely to engage in prevention practices than their single counterparts (aOR, 1.50). Additionally, men with a college education or higher were more likely to practice prevention than those with a middle school education or less (aOR, 2.28). For women (Figure 1B), both awareness (aOR, 1.23) and practice of prevention (aOR, 1.67) increased with age. Similar to men, married women had higher practice rates than single women (aOR, 1.55). Women living in mid-sized cities also had lower practice rates compared to those in urban areas (aOR, 0.78). However, unlike the findings for men, there was no significant difference in practice rates based on education level for women.

**Figure 2.**
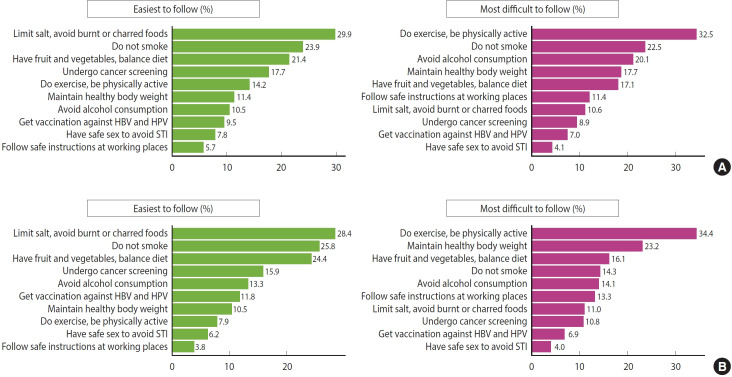
Rates of recommendation selected as easiest and most difficult to follow (A) men, (B) women. HBV, hepatitis B virus; HPV, human papillomavirus; STI, sexually transmitted infection.

Figure 2A displays the cancer prevention practices that men found easiest and most difficult to implement, while Figure 2B depicts the same for women. The perception of what is easy or difficult varies among individuals and can act as either barriers or motivators for engaging in these behaviors [18]. In this context, it is important to understand the ways in which the public perceives cancer prevention practices as easy or difficult to follow.

“Reducing salt intake and avoiding burnt or charred foods” was the recommendation most easily followed by both men (29.9%) and women (28.4%). This was followed by “no smoking and avoiding secondhand smoke” (23.9% men, 25.8% women) and “consuming a sufficient amount of fruits and vegetables and maintaining a balanced diet” (21.4% men, 24.4% women). Conversely, the most challenging guideline was “engaging in regular physical activity (i.e., at least 30 minutes, 5 days a week)” for both men (32.5%) and women (34.4%). For men, this was followed by “no smoking and avoiding secondhand smoke” (22.5%) and “limiting alcohol consumption” (20.1%). For women, the most difficult guidelines were “maintaining a healthy body weight” (23.2%) and “consuming a sufficient amount of fruits and vegetables and maintaining a balanced diet” (16.1%).

**Figure 3.**
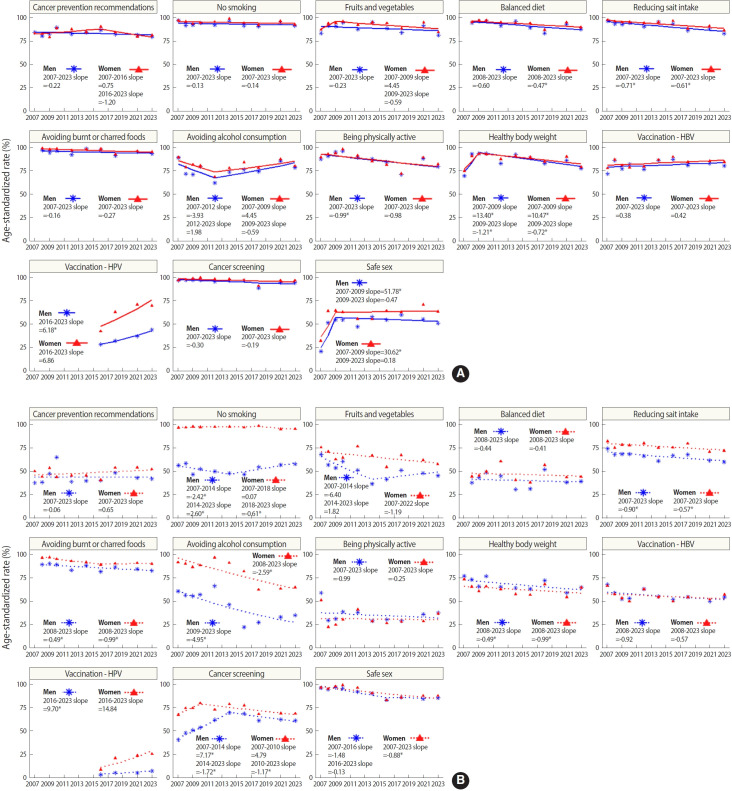
(A) Awareness and (B) practice of each cancer prevention recommendation, 2007-2023. HBV, hepatitis B virus; HPV, human papillomavirus. *p<0.05.

To examine changes in awareness and practice rates for each recommendation over time, we conducted a joint point analysis using data pooled from 2006, the year the survey was first conducted, through to the most recent data available from 2023. Figure 3A illustrates the temporal changes in awareness rates for each prevention practice, broken down by gender.

The awareness rates for both men and women were notably high, although there was a slight downward trend in most recommendations. Specifically, the recommendation to “reduce salt intake and avoid burnt or charred foods” experienced a significant decline in awareness among both genders. Awareness of “limiting alcohol consumption” decreased from 2007 to 2012 for both men and women, before showing an upward trend starting in 2012, though this change was not statistically significant. Furthermore, awareness of “engaging in regular physical activity (i.e., at least 30 minutes, 5 days a week)” significantly decreased among men. Similarly, awareness of “maintaining a healthy body weight” significantly declined for both men and women, although it had increased until 2009. Additionally, the gap in awareness of “immunization against HBV and HPV” between men and women was larger compared to other practices, with women showing significantly higher awareness levels.

Furthermore, Figure 3B illustrates the changes in rates for each prevention practice over time, segmented by gender, revealing a clear difference between men and women. For the practice of “no smoking and avoiding secondhand smoke,” women maintained a nearly 100% adherence rate from 2007 to 2023, though there was a notable decline after 2018. Men, in contrast, consistently demonstrated lower rates, but exhibited a significant positive trend beginning in 2014. National statistics suggest that the smoking prevalence among women in Korea falls between 6% and 7% [19]. However, this figure likely underestimates the true prevalence, influenced by the negative social perceptions of smoking. Given that this study was conducted through face-to-face interviews, there is a possibility that respondents may not have answered truthfully about their smoking habits due to its socially undesirable nature. Additionally, the observed decrease in prevalence since 2018 might also indicate an unreported rate of smoking.

Women’s practice of “consuming a sufficient amount of fruits and vegetables and a balanced diet” continued to decline, while men’s practice of the same has shifted from a decline to an increase, although not significantly, since 2014. For “reducing salt intake and avoiding burnt or charred foods,” there was a significant decrease in practice for both men and women. Although many people understand that high sodium intake is a major risk factor for cancer, other diseases, and obesity, and recognize the need to reduce sodium in their diets, our research indicates a significant decline in both awareness and practice of sodium consumption reduction among both genders. The WHO recommends limiting sodium intake to less than 2,000 mg/day and adopting a low-sodium diet [20]. Korea’s sodium intake was very high in 2012, at 4,549 mg, but has steadily decreased since then to 3,038 mg in 2021 [19].

“Limiting alcohol consumption” shows a significant decrease in practice for both genders, with a notably steeper decline among men. Men not only engaged in this preventive measure less frequently than women but also exhibited almost double the rate of decline. Given that alcohol consumption is a major risk factor for cancer, this trend of low and declining adherence to alcohol limitation is concerning from a cancer prevention standpoint. For “Immunization against HBV and HPV,” practice rates among both men and women have increased since the survey’s inception in 2006. The HPV vaccine is effective in preventing over 90% of major diseases caused by HPV infections, including cervical cancer. In Korea, the Korea Disease Control and Prevention Agency provides free HPV vaccinations for women adolescents aged 12-17 years and low-income women aged 18-26 years starting in 2024. This support likely contributes to the steady increase in vaccination rates. However, the practice rate remains low, possibly due to the restriction of the vaccine to women within this specific age group. “Undergoing regular cancer screening” has seen a significant decline since 2014. Similarly, “engaging in safe sex (i.e., having a single sexual partner and using condoms)” also decreased.

## DISCUSSION

The results of this study indicate a high level of awareness of the 10 recommendations for cancer prevention introduced in Korea, likely due to various publicity campaigns and public awareness efforts. However, the actual practice of cancer prevention has not yet aligned with the expected trend. Specifically, this study revealed a significant gender gap in practice (Supplementary Material 1), suggesting that while men are well aware of the health behaviors necessary for cancer prevention, they are not sufficiently implementing these practices. It is widely recognized that health behaviors and their underlying causes vary significantly by gender [1,21-23]. Many men may perceive themselves as healthy, take pride in their health, and consequently neglect healthcare due to the societal expectations of “masculinity.” The social norms that encourage men to take risks and demonstrate their strength significantly contribute to risky behaviors such as drinking and smoking [24].

This was also evident in the responses regarding which practices were the most difficult to adhere to. Men are more likely to report difficulty in following the recommendation to “limit alcohol consumption.” The trend in actual alcohol abstinence rates is alarming: in 2007, the rate of adherence to alcohol limitation was 60.5%, but by 2023, it had dropped to 34.8%. This decline can be attributed to a combination of gender characteristics and the socio-cultural environment. Korea has a very tolerant culture towards alcohol consumption. Unlike other risk behaviors, there is no significant negative public perception of alcohol consumption. Furthermore, the marketing and promotion strategies of alcohol companies are diverse and increasingly aggressive, often involving celebrities in their advertisements, while drinking regulations in the country are minimal. Many Koreans are unaware that alcohol is a class 1 carcinogen [25]. Therefore, urgent measures such as providing information on the harms of drinking, as well as regulating drinking and alcohol purchases, are required to create lasting change in the alcohol consumption trends in the country.

For the practice of “no smoking and avoiding secondhand smoke,” men reported finding it both easy and challenging. This task may not pose a difficulty for men who have never smoked or who have successfully quit, but it can be problematic for current smokers. Although numerous smoking cessation policies exist, such as cigarette pricing strategies, smoking bans, and restrictions on tobacco purchases, further consideration and support are necessary to facilitate the quitting process for smokers. Smoking is a potent carcinogen; thus, more stringent regulations and policies are essential.

Although women generally exhibit better health behaviors than men (Supplementary Material 1), our research identifies single women as a particularly vulnerable group. The composition and characteristics of single-person households vary significantly by gender and are not uniform across different age groups. Previous research has indicated that women living alone are more prone to engage in health-risk behaviors, such as increased alcohol consumption, and are less likely to participate in health-promoting activities like exercise, getting adequate sleep, and attending health screenings [26,27]. Despite this, the distinct socioeconomic and health characteristics of woman single-person households have not been adequately addressed. In light of these findings, it is crucial that policy interventions are tailored to address the health behaviors of women in single-person households.

This study also revealed important implications for cancer prevention practices. First, both men and women reported that reducing salt intake and avoiding burnt or charred foods were the easiest measures to adopt. However, despite this, the long-term trend shows a significant decrease in the practice of these behaviors. Reducing salt intake is widely recognized as a method to manage chronic diseases such as hypertension and diabetes. Similarly, avoiding burnt or charred food, which is a potent carcinogen, should be relatively straightforward given the widespread awareness of its risks through various campaigns, education, and publicity. Nevertheless, the adherence to these practices has significantly declined and remains above the levels recommended by the WHO. Considering the rising popularity of highly stimulating foods like *maratang* and *tteok-bokki* among Korean youth, it is necessary to continue promoting awareness about sodium intake and its health implications.

In addition to gender differences, this study also found that age, marital status, education, and residential area significantly impact cancer prevention awareness and practices. As previously mentioned, younger individuals are less likely to be aware of and engage in cancer prevention, perceiving it as a disease associated primarily with older age. However, lifestyle significantly influences cancer risk, with research suggesting that up to 50% of cancers could be prevented through lifestyle modifications [6]. Lifestyle habits formed early in life play a crucial role in cancer development, particularly as the incidence of early onset cancer—diagnosed in individuals under 50—continues to rise [28-30]. This trend highlights the importance of increasing awareness and promoting healthy behaviors among the youth. On another note, previous studies have indicated that a person’s place of residence correlates with health outcomes [31,32]. While the central government should spearhead large-scale cancer prevention policies, local governments possess a deeper understanding of the healthcare characteristics and needs specific to their communities based on city size. It is therefore crucial to empower local authorities [33]. Therefore, in mid-sized cities, unlike in urban and rural areas, localized and detailed management plans should be developed in close collaboration with the central government to enhance the adoption of healthy lifestyle practices.

This study builds on the analysis conducted up to 2021, which examined the overall awareness and practice behaviors related to cancer prevention from 2007 to 2021 [34]. The previous research provided a foundational understanding of trends in cancer prevention among the population. In this current study, recent survey results from 2023 have been incorporated, revealing differences in characteristics based on gender. This adds a new dimension to our understanding of cancer prevention behaviors, highlighting potential variations between men and women.

This study has limitations due to its cross-sectional survey design and reliance on self-reported data, which may introduce bias. To mitigate this issue, we conducted a face-to-face survey with a sample size of 4,000 to minimize response bias and enhance statistical reliability and accuracy. Additionally, by performing a time-series analysis of previous iterations of the same survey, we aimed to address the limitations inherent in cross-sectional surveys by tracking changes in cancer prevention awareness and practice rates over time.

Based on the results of this study, we anticipate identifying specific intervention points for cancer prevention practices and developing more effective cancer prevention policies in the future. Additionally, we expect that promoting healthy lifestyle habits for cancer prevention will contribute to curbing the rise in cancer incidence.

## Figures and Tables

**Figure f4-epih-47-e2025003:**
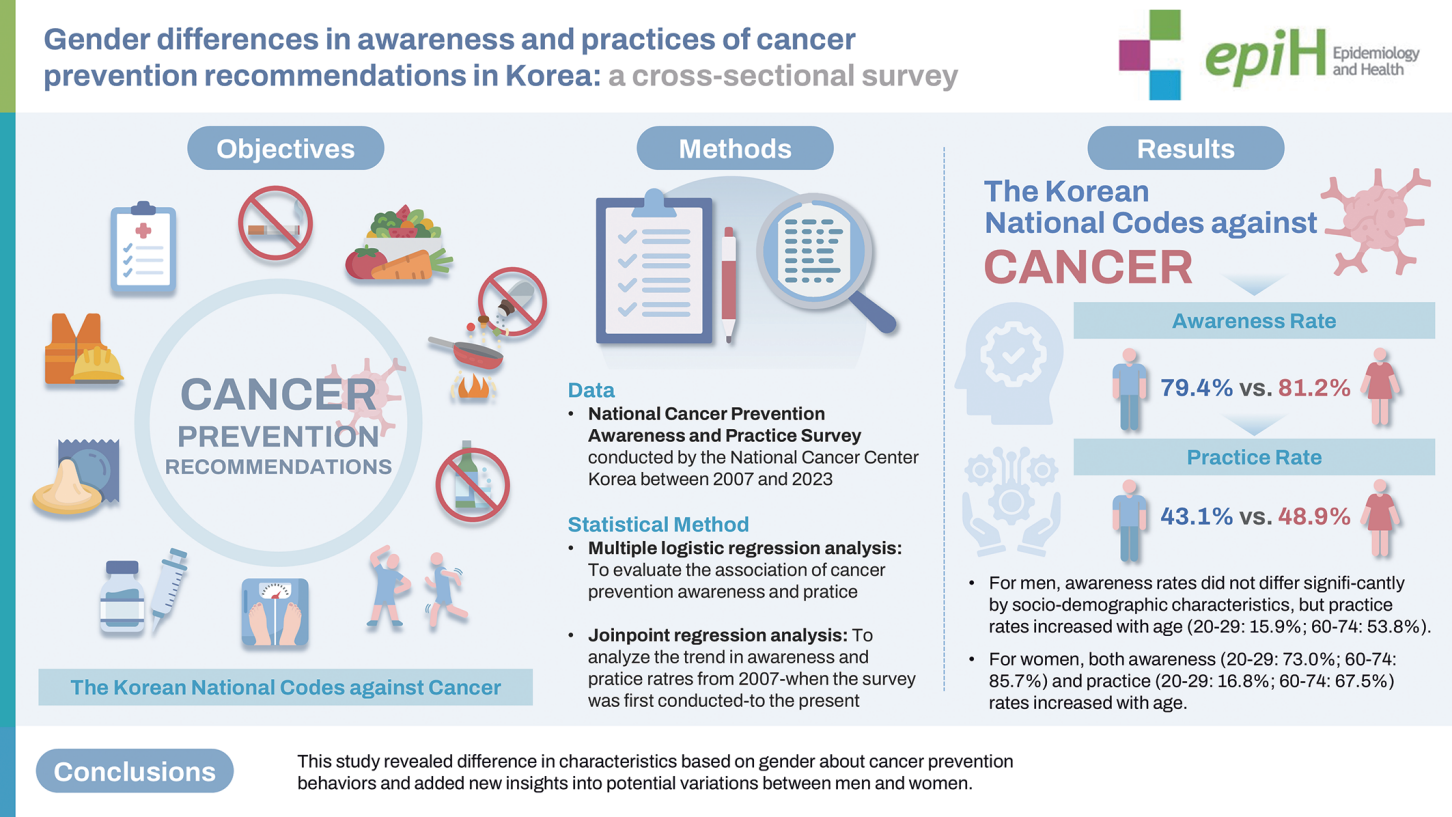


**Table 1.** The awareness and practice of cancer prevention according to the general characteristics of the study participants in the Cancer Prevention Awareness and Practice Survey in 2023

Characteristics	Men	Women	Awareness				Practice			
			Men	p-value	Women	p-value	Men	p-value	Women	p-value
All	2,027 (50.7)	1,973 (49.3)	79.4		81.2		43.1		48.9	
Age (yr)				0.910		<0.001		<0.001		<0.001
20-29	333 (16.4)	304 (15.4)	78.4		73.0		15.9		16.8	
30-39	348 (17.2)	323 (16.4)	79.0		78.9		37.9		36.8	
40-49	413 (20.4)	401 (20.3)	79.7		83.3		46.2		52.6	
50-59	444 (21.9)	435 (22.0)	80.9		81.6		52.9		55.2	
60-74	489 (24.1)	510 (25.8)	78.7		85.7		53.8		67.5	
Region				0.030		0.720		0.030		0.010
Urban	866 (42.7)	881 (44.7)	78.1		80.6		45.8		50.7	
Mid-sized cities	1,003 (49.5)	933 (47.3)	81.5		82.0		40.2		45.8	
Rural	158 (7.8)	159 (8.1)	73.4		80.5		46.8		57.2	
Marital status				0.190		<0.001		<0.001		<0.001
Single	657 (32.4)	543 (27.5)	77.0		75.9		27.9		27.4	
Married	1,312 (64.7)	1,345 (68.2)	80.6		83.6		50.5		57.1	
Widowed/separated/divorced	58 (2.9)	85 (4.3)	79.3		77.6		48.3		56.5	
Education Level				0.130		0.330		0.140		<0.001
Middle school or lower	63 (3.1)	126 (6.4)	71.4		85.7		44.4		64.3	
High school	583 (28.8)	691 (35.0)	77.9		80.2		46.5		54.3	
College or higher	1,381 (68.1)	1,156 (58.6)	80.4		81.4		41.6		44.0	
Monthly income (million Korean won)				0.030		0.850		0.340		0.810
<3	334 (16.5)	367 (18.6)	74.3		82.3		44.0		49.6	
3-6	1,220 (60.2)	1,109 (56.2)	79.8		81.0		44.0		49.2	
≥6	473 (23.3)	497 (25.2)	81.8		81.1		40.2		47.7	

Values are presented as number (%) or percentage.

## Supplementary materials

Supplementary Material 1. Chi-Square analysis of gender differences each cancer prevention recommendation
