# Supplementary material for: Gender differences in awareness and practices of cancer prevention recommendations in Korea: a cross-sectional survey
Source: Epidemiol Health. 2025 Jan 15;47:e2025003. doi: 10.4178/epih.e2025003 (PMC11920676; doi:10.4178/epih.e2025003)
Supplement: Supplementary Material 1. — Chi-Square analysis of gender differences each cancer prevention recommendation [file epih-47-e2025003-Supplementary-1.docx]

**Supplementary Material 1. Chi-Square analysis of gender differences each cancer prevention recommendation**

| **Prevention recommendation** | **Outcome** | **Gender** | **N (%)** | **value** | **P-value** |
| --- | --- | --- | --- | --- | --- |
| **Cancer Prevention Recommendation** | **Awareness** | Men | 1,609 (79.98) | 2.090 | 0.148 |
|  |  | Women | 1,603 (81.25) |  |  |
|  | **Practice** | Men | 874 (43.12) | 13.274 | < 0.001 |
|  |  | Women | 965 (48.91) |  |  |
